# Scared, powerless, insulted and embarrassed: hesitancy towards vaccines among caregivers in Cavite Province, the Philippines

**DOI:** 10.1136/bmjgh-2021-006529

**Published:** 2021-09-02

**Authors:** Jhoys Landicho-Guevarra, Mark Donald C Reñosa, Jonas Wachinger, Vivienne Endoma, Mila F Aligato, Thea Andrea Bravo, Jeniffer Landicho, Kate Bärnighausen, Shannon A McMahon

**Affiliations:** 1 Department of Epidemiology and Biostatistics, Research Institute for Tropical Medicine, Muntinlupa City, Metro Manila, Philippines; 2 Heidelberg Institute of Global Health, Ruprecht Karls Universität Heidelberg, Heidelberg, Baden-Württemberg, Germany; 3 School of Public Health, University of the Witwatersrand, Johannesburg-Braamfontein, Gauteng, South Africa; 4 International Health Department, Johns Hopkins University Bloomberg School of Public Health, Baltimore, Maryland, USA

**Keywords:** vaccines, immunisation, public health, child health, health policy

## Abstract

**Background:**

Several studies have highlighted that vaccine hesitancy (VH) is among the most important threats to global health, especially in low- and middle-income countries, including the Philippines. However, there is a dearth of literature exploring family experiences of—or concerns related to—childhood vaccinations that gives voice to vaccine hesitant caregivers (VHCs) of small children. Here, we present insights from VHCs from the Philippines.

**Methods:**

This research draws on in-depth interviews (IDIs) with 44 VHCs who had previously delayed or refused vaccination for their children in rural and urban communities in Cavite Province, the Philippines. Amid the COVID-19 pandemic, we conducted IDIs via an online platform of the respondents’ choosing (ie, Facebook messenger call, Skype and Zoom). All interviews were recorded, transcribed, translated and analysed drawing from the tenets of constructivist grounded theory. We use the social ecological model to structure our results.

**Results:**

Among the reasons for delay or refusal of childhood vaccinations, a fear of side effects emerged as the most salient concern, exacerbated by previous negative experiences (including trauma) from a dengue vaccine controversy in 2017. Respondents cited the dengue vaccine controversy as they expressed reluctance (regarding any new vaccines) and suspicion (towards school-based vaccination, the distribution channel used for the dengue vaccine). Heads of households opposing vaccines, perceptions that vaccines are non-essential and influences from the social and traditional media or neighbours contributed to further refusal and delay. Upon probing, VHCs recounted health system concerns particularly with regards to healthcare workers who are sometimes unable to answer their questions and can be dismissive or disrespectful regarding caregivers’ concerns.

**Conclusion:**

Understanding VH from the lens of VHCs highlights pathways for interventions to regain trust and bolster confidence towards vaccines. Our findings may serve as linchpins in the development of VH interventions aiming at changing behaviour on a population scale.

**Key questions**

**What is already known?**

- Studies examining factors that contribute to vaccine hesitancy remain limited, are largely quantitative in nature, and mostly stem from high-income countries.
- Vaccine misinformation and disinformation has been pervasive and entrenched, causing caregivers across countries to lose trust and confidence in long-established childhood vaccinations.
- This development and dearth of information is particularly salient in the Philippines, where a highly politicised vaccination controversy has recently resulted in declines in vaccine confidence.

**What are the new findings?**

- Respondents described drops in vaccine uptake as a by-product of the Dengvaxia controversy and the subsequent spread of misinformation via social and traditional media. Vaccine hesitant caregivers (VHCs) described acute concerns related to ‘new vaccines’ as compared with those with a long-standing history.
- VHCs often drew comparisons between today’s ‘overly vaccinated children’ who seem more prone to sickness compared with children in past generations who were less vaccinated.
- VHCs’ dissatisfaction with healthcare workers (HCWs) emerged as a driving factor of vaccine hesitancy. Generally, VHCs felt that nobody is beholden or responsible for their vaccination concerns, and in instances when they had raised hesitations with HCWs, they described feeling dismissed, unheard and unseen.

**What do the new findings imply?**

- Our findings highlight the enduring effects of misinformation and disinformation associated with politicised vaccine controversies, including their impact on the roll-out of new vaccines.
- The need for coordinated action in refining current vaccination campaigns and development of more interventions based on empathy, regaining trust and engaging with concerned parents/caregivers in a respectful manner are paramount.

## Background

Vaccine confidence is decreasing in several countries across the globe, particularly in a number of low- and middle-income countries (LMICs), despite the instrumental role of vaccination in preventing deaths and disabilities among millions of children annually.^1–4^ In 2019, the World Health Organization (WHO) named vaccine hesitancy (VH) among the top ten threats to global health.^5^ In LMICs, in addition to weaker health systems and often constrained hospital access, a decline in vaccination rates can—within a short period of time—result in outbreaks of previously controlled or domestically eliminated vaccine-preventable diseases such as measles and polio.^6–9^

Within the last decade, a considerable amount of quantitative literature has outlined the complex determinants that underpin parental vaccine decision making.^10–12^ Qualitative systematic reviews in 2016 and 2020 highlighted parental alternative health beliefs (ie, natural and organic living) and mistrust towards vaccine-related institutions as reasons why parents refuse or delay vaccination of their children.^13–15^ Other reasons include low perceived vaccine safety and efficacy, low levels of trust in the government and low perceived susceptibility to vaccine-preventable diseases.^10^ Recent studies also highlighted parents’ vaccine reluctance due to fears of serious side effects and long-term adverse events (ie, development of disability).^16–19^ In addition, several studies underscore that parents struggle to reconcile conflicting information from healthcare workers (HCWs), religious leaders, the internet or social media and their social circles (ie, family members, neighbours), which complicates vaccination uptake.^11 12 20–22^

A 3-year WHO/United Nations International Children’s Emergency Fund (UNICEF) global project confirmed that VH is present in a majority of countries globally.^23^ However, with most research originating in high-income countries (HICs), evidence to guide policymakers from LMICs considering VH interventions is limited.^24 25^ There is a paucity of data from LMICs describing the reasons why parents or caregivers refuse or delay childhood vaccinations. Such insights can help us to better understand VH in specific contexts (ie, acknowledging social, cultural and geographical variations) to ensure interventions are well directed and received. To address this gap, we focus on the case of the Philippines to explore concerns of and barriers faced by urban and rural vaccine hesitant families in the country. With these findings, we seek to inform the development and refinement of VH interventions, theory development and derivation of pathways of care delivery for VH families.

## Methods

### Study design and setting

This study is part of a larger mixed methods study (‘Project SALUBONG’) aiming to develop an intervention that draws on vaccination narratives and imagery from caregivers of under-5 children and HCWs in the Philippines.^26^

The Republic of the Philippines, the setting of this study, is an archipelago in Southeast Asia consisting of more than 7000 islands divided into 17 administrative regions.^27^ While the Philippines was once among the countries with high vaccine confidence and uptake, vaccine confidence rapidly declined in 2018 following the large-scale, highly politicised Dengvaxia vaccine scare. Dengvaxia was a novel dengue vaccine that was first introduced as part of a national school-based immunisation programme, only to be abruptly pulled from the market following safety concerns for children who previously had not been infected with dengue.^28 29^ As a consequence, VH, general vaccine safety perceptions, and trust in the government among Filipino parents declined,^29–31^ and plummeting vaccination rates resulted in outbreaks of previously controlled or domestically eliminated diseases such as measles and polio.^32 33^ While recent data shows that vaccine confidence and uptake are exhibiting first signs of recovery,^1^ restricted access to healthcare facilities has persisted due to natural disasters (ie, a volcanic eruption in 2020) and the ongoing COVID-19 pandemic.^34–36^

This chain of events has repeatedly been marked by the exacerbated role of misinformation or opposing narratives in online media, particularly social media. In the context of the Dengvaxia scare, rumours and fear narratives (such as unverified rumours and claims about child deaths) rampantly spread on social media platforms (particularly on Facebook and Twitter) creating social divisiveness and public tension.^37^ The Philippines, where a majority of the population in urban and rural areas accesses the internet via Facebook, and more than 93% use social media (ie, YouTube and Facebook) on a daily basis,^38^ is a promising case study to investigate individual vaccination narratives and barriers.

**Figure 1:**
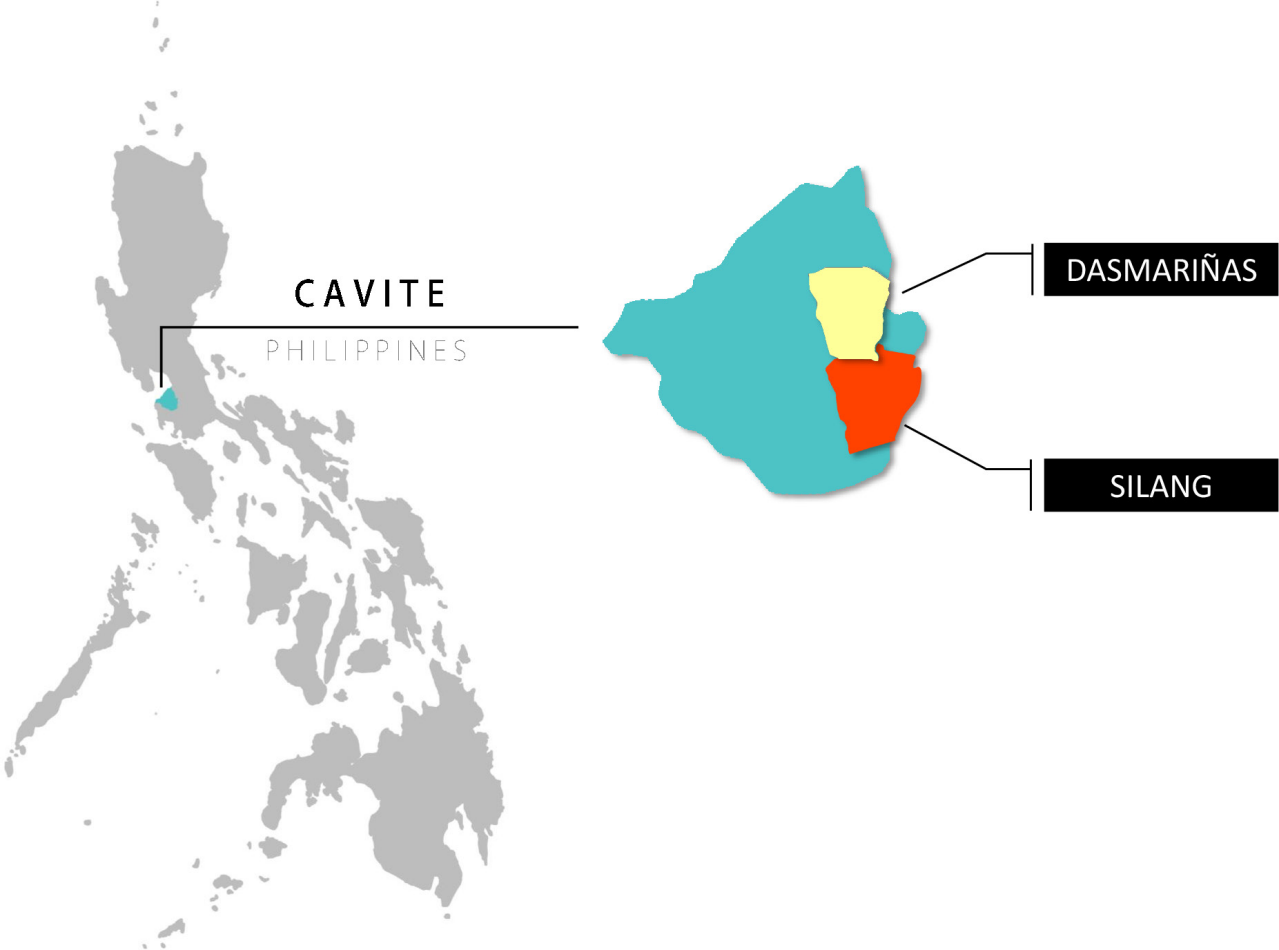
Map of Cavite, Philippines showing the two study sites, Dasmarinas City and the Municipality of Silang (map credit: courtesy of www.mapchart.net).

Within the Philippines, we purposively selected the Calabarzon region, which is the most populous region in the Luzon group (est. population 14 million) and a microcosm of the modern Philippines in terms of sociodemographic status, religious makeup, and health facility and family household structures (see figure 1).^39^ From 2018 to 2019, Calabarzon saw a 300% increase in measles cases.^40–42^

### Recruitment and data collection

As part of the larger project SALUBONG,^26^ we purposively selected vaccine hesitant caregivers (VHCs) of under-5 children who had not completed their vaccination according to the recommended schedule in the preceding years (from 2018 up to the date of collection). Eligibility in terms of vaccine uptake was determined based on vaccination records provided by HCWs in the region. Caregivers (ie, mothers, fathers, grandparents) whose records showed delays or refusals of at least one childhood vaccine were invited to participate. We recruited the caregivers in partnership with local HCWs. HCWs initially visited the identified respondents at their homes, provided them with study information sheets and consent forms, and informed them that a member of the research team would call them to further explain the study and answer any questions that they might have. If potential respondents agreed to participate, we set an appointment for an interview at a time via a platform of their choosing, and we transmitted free Internet mobile data packages to ensure connectivity. Additional information regarding the exact study procedures, including phone scripts for participant recruitment, is published elsewhere.^26 43^ Ethnicity, race, political orientation, religion and class were not criteria for inclusion or exclusion. Eligibility criteria included living in the designated study area and being at least 18 years old or an emancipated minor (15–17 years old but with under-5 children). Incapacitated persons were excluded.

**Figure 2:**
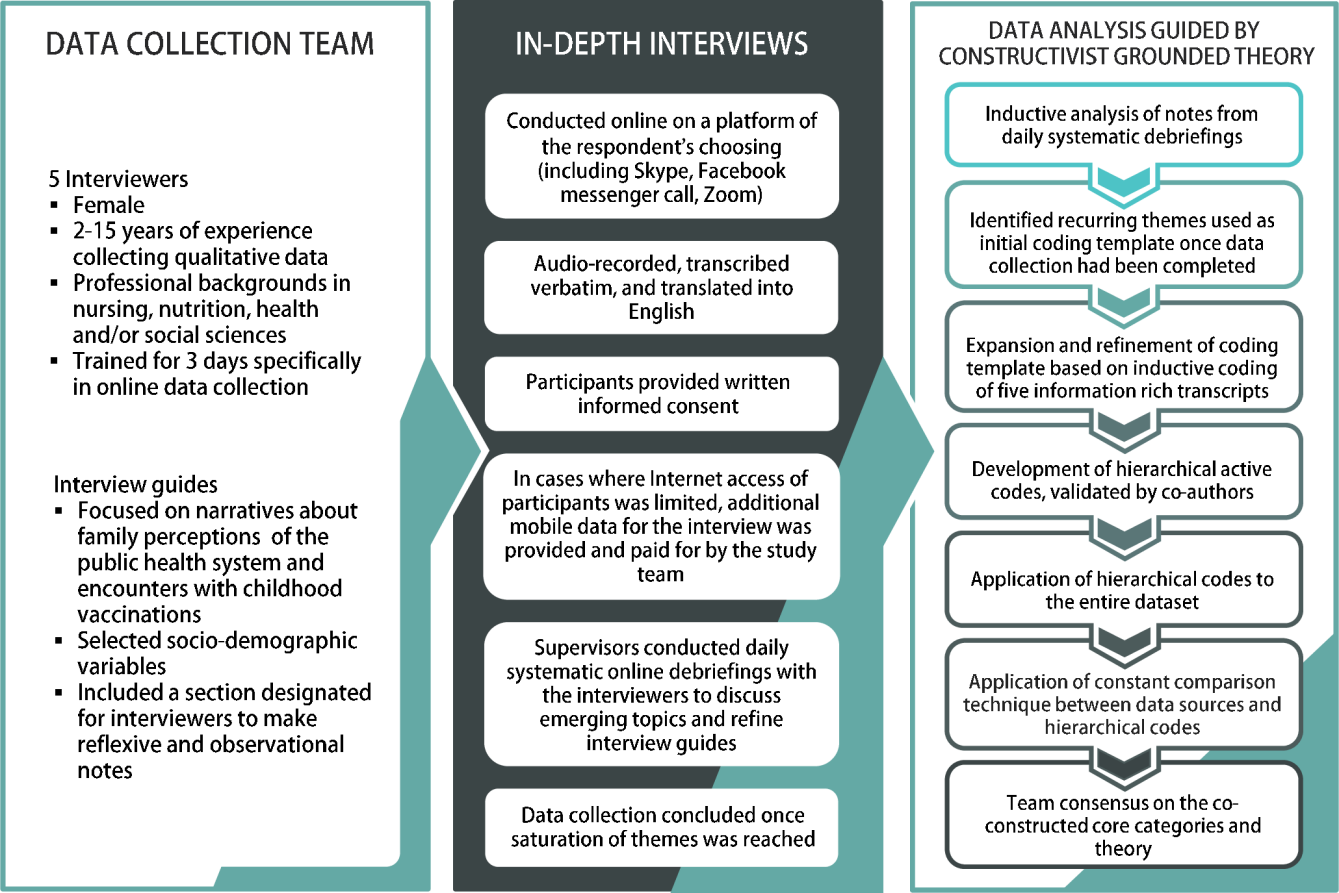
Description of study’s recruitment, data collection and analysis.

Between August 2020 and March 2021, we conducted semistructured in-depth interviews (IDIs) using open ended questioning techniques (see figure 2). A team of five interviewers were trained to collect qualitative data using standardised instruments. Our detailed experience and learnings from switching to online IDIs (we shifted to this approach amid the COVID-19 pandemic lockdown) are described elsewhere.^43^

### Data analysis

We drew on constructivist grounded theory (CGT) as our analytical framework.^44^ We began our inductive approach during data collection with analysis of our notes from the systematic debriefings.^45^ These team debriefings were essential to compare findings, discuss differences and triangulate data, determine agreement on the direction of the analysis (see figure 2), and to develop a preliminary codebook. This codebook was applied to five information rich transcripts and further refined. By the time data collection was completed, we identified several reoccurring themes and finalised our codebook. The final codebook was manually applied to the full dataset in an iterative process with continued openness to new codes that emerged inductively at later stages of coding. Three phases of coding—open, axial and selective—allowed the team to develop and connect emerging subthemes and structure the narratives into categories of information.^44^ Analysis of raw data in the form of observational and reflexive notes was conducted alongside these phases of coding to ensure the co-constructed narrative of both the respondents and interviewers was embedded within our codebook.^44^ We used coding language that was active and as close to the respondents’ terms and phrases as possible to ensure we were co-constructing accurate categories of information.^44^ We then reviewed the categories of information and regrouped them into concepts. This process developed our understanding of the relationships between codes and the co-construction of concepts. We applied a constant comparison technique between our data sources and coding to develop focused codes, which formed our core categories and the basis of our theory development. During debriefings, the team agreed that barriers to vaccination aligned with tiers of the social ecological model (SEM).^46^

### Reflexivity

The lead authors (JLG and MDR), both with a background in nursing, acknowledge the personal preconceptions and contextual experiences in the implementation of the vaccination programme that may influence the way this data was interpreted and coded.^47^ More so, their work at the Research Institute for Tropical Medicine focuses on childhood illnesses in the rural and urban provinces of the Philippines, which in some way or another exposed them to the health system barriers and issues faced by Filipino caregivers.

These experiences shaped how the lead authors viewed and coded the data. As they reflect on their experiences, they understood that the way we perceive the world is different from others and that reality is subjective and multiple. We believe that drawing on CGT allows to provide a direction to highlight and explicate the richness, breadth, and depth of the experiences of both the respondents’ and our own shared experiences because of CGT’s emphasis on co-construction of experiences.^48^ Further, our hands-on experiences of the barriers faced when delivering health services in primary health facilities in the study setting shaped our view of the data and thus the manner in which we position the findings. When a spectrum of barriers across different layers emerged during coding, we decided to use an SEM as our theoretical lens to allow us to identify challenges and articulate how these affect the delivery of vaccination services across levels as a means to offer guidance not only for other scholars, but also for health promoters in the field.^46^ We engaged in a creative process of theory construction (both inductively and deductively) and used other analytical tools such as abduction, which involves engaging with intuitive and creative ideas that help to explain unanswered or unexpected observations.^44 49^

### Patients and public involvement

Patients and/or the public were not directly involved in the design, recruitment, conduct, reporting or dissemination plans of this research; their only involvement is as research participants.

## Results

We approached 55 VHCs of children under-5 and completed 44 interviews, 11 respondents refused to participate. Reasons included busy schedules (n=2) and that husband or other family members did not allow participation (n=4); and, some did not provide specific reason (n=5). Table 1 shows the demographic characteristics of the respondents. A majority of respondents were female, younger than 40, and had at least a high-school degree (table 1).

**Table 1:** Demographic profiles of the respondents

Characteristics	(n=44)	%
Sex		
Male	2	4.55
Female	42	95.45
Age group		
<30 years	17	38.64
30–40 years	16	36.36
41–50 years	8	18.18
>51 years	3	6.82
Number of children		
1–2	15	34.09
3–4	16	36.36
5–6	8	18.18
7–8	2	4.55
9–10	2	4.55
11	1	2.27
Number of children under-5		
1	23	52.27
2	17	38.64
3	3	6.82
4	1	2.27
Highest educational attainment		
None	2	4.54
Primary	10	22.73
High school	24	54.55
College	7	15.91
Vocational training	1	2.27

**Figure 3:**
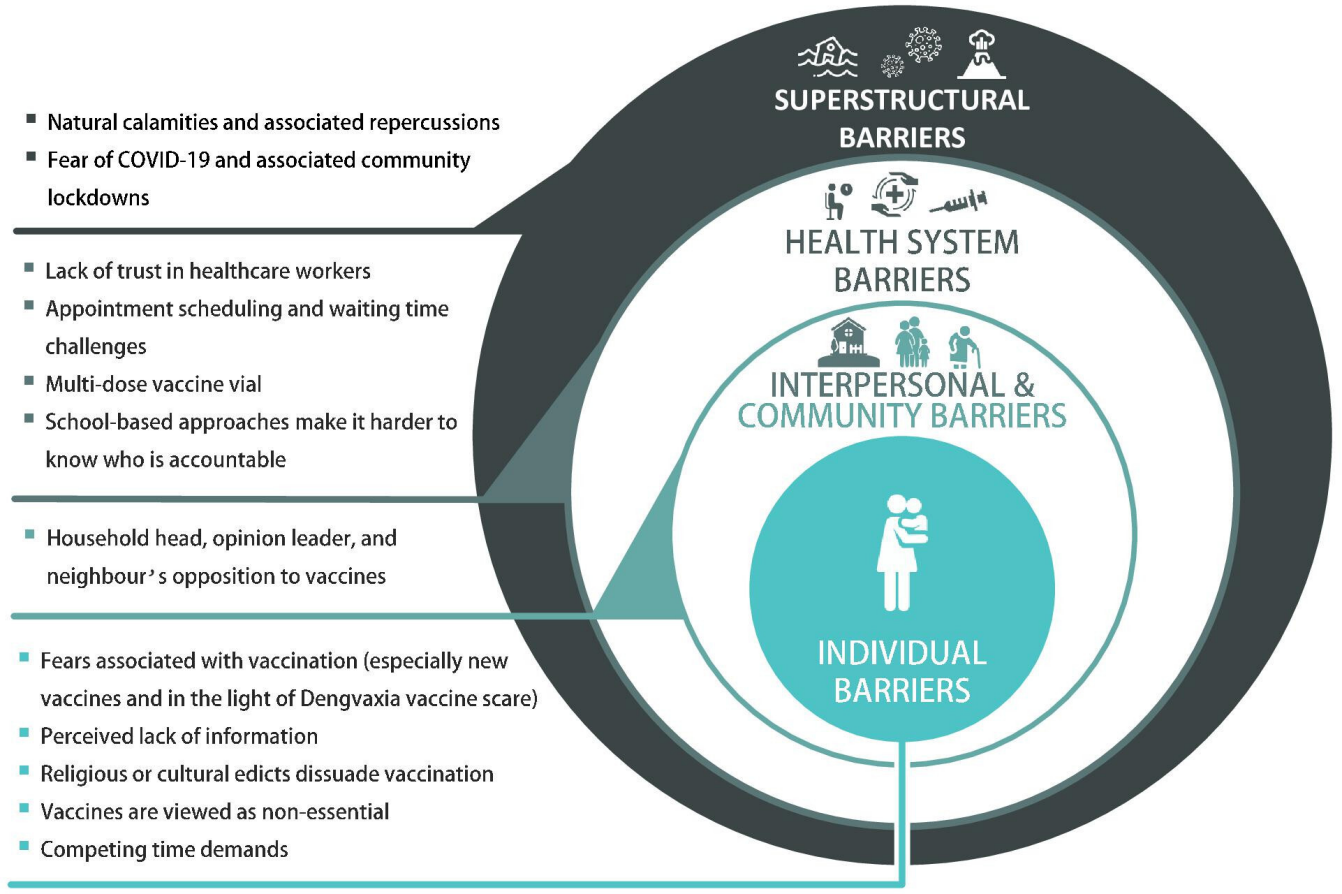
Barriers of childhood vaccine uptake in the Philippines (icons credit: courtesy of www.flaticon.com).

To highlight the complex reasons of VHCs evident in our findings, we present the results within the SEM framework (see figure 3). Individual barriers emerged as the most salient from the data, we therefore present the results from the central layer of the SEM outwards. For each theme, we present salient quotes in table 2. After each quote, we provide respondents’ number of children and age as identifiers in parentheses.

**Table 2:** Parental factors that contribute to vaccine hesitancy in the Philippines

Themes	Illuminating quote
**Individual barriers**	
Fears associated with vaccination (especially new vaccines and in the light of Dengvaxia vaccine scare)	“When my child experienced fever after vaccination I felt guilty for why I submitted my child for vaccination(…)because I am not used to my child getting sick(…)I felt fearful and confused too.” (Mother of 2, age 25)
	“Our second child was injected by Dengvaxia when at school, that was what we worried about. We learned from the television that many have died from Dengvaxia. This is the reason why we do not want our child to be vaccinated again(…)even the youngest one because we are worried for our child’s safety.” (Father of 3, age 30)
	“(…)I felt nervous with the new vaccines, but with the old vaccines until measles, I am fine as I do not have any bad experience.” (Mother of 4, age 38)
Perceived lack of information	“Health workers will visit our houses, informing us to bring the child at the health center for vaccination…no other information but name of vaccine and doses only… no information on advantages or disadvantages…or the side effects to be expected.” (Mother of 7, age 46)
Religious or cultural edicts dissuade vaccination	“Our belief is that we are made by God naturally, so it is also a need that what enters our body is also natural- not the chemicals that once ingested(…)will have side effects in the body.” (Mother of 10, age 46)
Vaccines viewed as non-essential	“It is not needed. My baby is already healthy and active.” (Mother of 3, age 25)
	“It is (*hindi uso*) not trendy and, that we turned okey even without injections.” (Mother of 5, age 28)
Competing time demands	“I was washing clothes during the day my child was scheduled for vaccination, and I remember it night time already(…)My husband was working and I was left alone at the house(…)I need to go to the market for our food(…).” (Mother of 6, age 33)
**Interpersonal and community barriers**	
Household head, opinion leader and neighbours’ opposition to vaccines	“My husband does not want my children to be vaccinated because they might get sick(…)and I will obey my husband’s decision because I don’t want to fight with him over our children’s health.” (Mother of 2, age 25)
	“(…)my father got mad when I let my child be vaccinated with polio vaccine, the child has developed fever and was crying overnight, he told me not to let my child be vaccinated again(…)my father knew what is right(…).” (Mother of 2, age 29)
	“My neighbors told me that vaccine is not good for my children because they will get infected with the disease, which also I believed, thus I am also hesitant for my children to be vaccinated.” (Mother of 2, age 25)
**Health system barriers**	
Lack of trust in HCWs	“(…)it’s hard to open up, when you have concerns in mind, and you want to clarify. The way she (healthcare worker) talks is different, very straightforward, she can hurt someone’s feelings, it’s like an insult(…)When you have follow-up questions, she shouts(…)with limited patience.” (Mother of 5, age 35)
Appointment scheduling and waiting time challenges	“It was difficult(…)health center is full of children(…)sometimes the healthcare worker is not yet there(…)sometimes we are asked to return because the vaccinator is not yet available(…)busy attending to childbirth(…). So, I decided not to go back because it was really far(…)and that same thing will happen.” (Mother of 5, age 31)
Multidose vaccine vials	Sometimes when we are scheduled for vaccination, there was no available open vaccine vial(…)they will need to reschedule us so that for the next vaccine vial that will be opened, there are also other children who will be vaccinated.” (Mother of 2, age 23)
School-based approaches make it harder to know who is accountable	“I did not allow my children to be vaccinated in school because if there are problems, like side effects, I do not know whom to approach, at least when it was given by the health center, I know them well and I can approach them in case there are problems.” (Mother of 3, age 40)
**Superstructural barriers**	
Natural calamities and associated repercussions	“It was difficult to walk going to the health center(…)sometimes it was raining, sometimes too hot and we had no umbrella(…)no transportation also, your option is to walk only(…)then there are roads that are elevated.” (Mother of 5, age 31)
Fear of COVID-19 and associated community lockdowns	“I do not want to risk bringing my baby to the health center because of COVID-19(…)they told me that I need to put a mask to my baby, how is that even possible? My baby is not used to wearing a mask(…)she can’t breathe” (Mother of 3, age 34)

HCWs, healthcare workers.

### Individual barriers

#### Fears associated with vaccination (especially new vaccines and in the light of Dengvaxia vaccine scare)

Respondents reported fears of side effects, especially based on things their children experienced or ‘horrible stories and videos’ (Mother of 7, age 46) they had heard and watched. Side effects feared the most were high fever, cough, and diarrhoea, especially as they saw their children being too small to handle such illnesses. Some respondents recalled their experiences with their first child, and were not prepared to manage those symptoms again, leaving them with feelings of guilt and confusion. Respondents described that their fears had further increased due to television news and social media posts about children getting sick and dying following a Dengvaxia vaccination, and the existence of ‘people pretending to be health workers’ (Mother of 7, age 46) who are ‘visiting houses to inject an unknown vaccine’ (Grandmother of 2, age 52). Respondents also shared that they remained afraid that vaccines advocated by the government might make their children sick, similar to children who had ‘died due to Dengvaxia’ (Mother of 3, age 40). One respondent shared that he continues to struggle with guilt about having had his child vaccinated with Dengvaxia, and that he would not want his other children to be vaccinated again as he is still ‘traumatized watching my child exhibiting side effects everyday’ (Father of 3, age 30).

Many respondents shared that they were in favour of ‘traditional or routine vaccines’ (Mother of 3, age 34) for small children that had been given in health centres for many years (ie, BCG, diphtheria, pertussis, tetanus (DPT), hepatitis, polio, measles). In contrast, ‘*bagong labas* (new vaccines)’ (Mother of 5, age 28), whether those newly introduced (such as the Dengvaxia vaccine) or anticipated to be introduced soon (such as COVID-19 vaccines, which were not yet available at the time of data collection), were perceived as more dangerous.

#### Perceived lack of information

Several respondents reported that they had not received detailed information on the recommended vaccines by HCWs, for example regarding ‘what could be the possible advantages and disadvantages’ (Mother of 7, age 46), or the side effects to be expected. The only information they had received were the name of a specific vaccine being injected to their children, the corresponding number of doses, and the expected date for the succeeding doses. A majority of respondents received no specific instructions regarding what to do in instances when a child may experience adverse events. Respondents felt that this information would be imperative for them to make the right decision for the safety of their children, especially in light of reports of suffering associated with Dengvaxia.

#### Religious or cultural edicts dissuade vaccination

Respondents shared fears that some of the vaccines’ ingredients (eg, pork-derived gelatin or any pork-products) might be incompatible with religious beliefs and notions of purity or hygiene. Respondents also described how vaccines represent a chemically based creation (‘a toxic mix’ (Mother of 10, age 46)) that conflicts with the body’s natural ability to address disease.

#### Vaccines are viewed as non-essential

Respondents often decided against vaccination for their children based on careful evaluation of their own experiences as children. Respondents felt that during their childhood, vaccines were viewed as ‘non-essential’ (Mother of 3, age 34) or ‘*hindi uso at hindi nakasanayan* (not trendy and not accustomed)’ (Mother of 2, age 25) and only used by a tiny minority, and that nevertheless the non-vaccinated majority grew into healthy adults. As one mother remarked: ‘So you can see that not having vaccines also works’ (Mother of 5, age 28). Respondents also added that today’s children often were overvaccinated and appear sickly or weaker than children in the past.

#### Competing time demands

Respondents, particularly mothers, described situations where they had working partners, how the hectic reality of doing household tasks led them to forget vaccination appointments or follow ups. One mother mentioned how by the time she had ‘finished all home tasks, so in the late afternoon’ it was already too late when she remembered her child’s vaccination appointment (Mother of 6, age 33).

### Interpersonal and community barriers

#### Household head, opinion leader and neighbours’ opposition to vaccines

Respondents, particularly mothers, said they had to defer to heads of households (husbands, mothers-in-law), and that this person routinely opposed vaccination because they believed it harms children. Mothers described a desire to obey their husbands’ orders as a means to avoid arguments, but also to avoid blame in the event that a vaccinated child becomes sick. Mothers also described how violating the will of a household head could lead to abandonment and, thus, destitution.

Oftentimes, respondents conveyed that they were living with their or their spouse’s parents, as their income is not sufficient to live on their own, and in this household arrangement they had to defer to the preferences of grandparents or those with financial clout in the family. One respondent shared an instance where she disobeyed her in-laws (who were opposed to vaccination), and had her child vaccinated against polio. This mother ultimately regretted the decision, as she was blamed when her child developed fever and cried throughout the night. Respondents also described neighbourly chatter that heavily emphasised the danger of vaccines, namely that vaccines ‘cause complications or side effects’ (Mother of 7, age 46), including child death. Respondents highlighted that these stories were credible as their neighbours, parents themselves, had experienced these side effects firsthand.

### Health system barriers

#### Lack of trust in HCWs

Poor patient–provider relationship emerged as a challenge in accessing childhood vaccination services. Respondents spoke about their preferences regarding who would administer vaccines. Some respondents voiced distrust towards HCWs’ skills to do the injection, fearing for their children’s safety, as one witnessed a HCW vaccinating children ‘using a swollen and bandage-covered arm’ (Mother of 5, age 30). Other respondents expressed a need for further information about vaccine safety and side effects but felt that HCWs were often dismissive of their concerns and instead ‘the health worker shouts and has limited patience’ (Mother of 5, age 35). Most respondents stated that they saw their questions to be valid as the health of their children was at stake, but felt insulted, hurt, and embarrassed. These stories prompted others not to come back, to prefer other HCWs, or to look for other health centres that would be more accommodating.

Some respondents also shared instances when they forgot or lost their vaccination cards and were afraid that HCWs would scold and shame them, as previously experienced by themselves or other parents. Such experiences also resulted in respondents preferring private facilities over government health centres. Respondents shared that in private facilities ‘they are being prioritized’ (Mother of 6, age 35) and that there would be medical doctors available, unlike in health centres which mostly are managed by one midwife.

#### Appointment scheduling and waiting time challenges

Respondents shared concerns regarding the facilities’ policy to vaccinate on a ‘first come, first serve’ basis rather than by appointment, which was seen as ‘unreasonable’ (Mother of 2, age 23) and ‘impossible’ (Mother of 5, age 31), citing competing time demands. Respondents mentioned that even if they arrived earlier, the waiting line for vaccination would already be long, as HCWs are busy attending to other health-related matters. This led to disappointment and irritation of respondents who travelled a long way only to end up being rescheduled to the next day or week, resulting in some parents deciding ‘not to return because the same waiting game might happen’ (Mother of 5, age 31). One respondent also claimed that her child was rescheduled—however, ‘a month had passed and still no follow-up date for vaccination was given’ (Mother of 2, age 19).

#### Multidose vaccine vials

Vaccines being packaged with multiple doses per vial was reported to be a problem, as it resulted in health centres only opening a new vial when they could be sure to have enough patients to avoid vaccine wastage. One respondent recalled an instance when the HCW rescheduled her child’s vaccination because there was ‘no available open vial during the time of our visit’ (Mother of 2, age 23).

#### School-based approaches make it harder to know who is accountable

Respondents expressed negative feelings toward school-based immunisation strategies employed by the government and lamented the school-based distribution of dengue vaccines. Parents felt left alone with their questions and were unsure who would be responsible after learning about the potential side effects. In turn, respondents preferred to receive vaccines at the health centre, where they are familiar with the HCWs and would know who would be responsible to administer care, reconcile concerns and resolve any longer-term issues in case a child developed side effects.

### Superstructural barriers

#### Natural calamities and associated repercussions

Erratic weather conditions (typhoons, severe storms) and their effects on communities (eg, washed out roads, flooded houses) were described as problematic because of the safety priority of the families. Although respondents mentioned that vaccines are freely available in health centres, access to such centres can be problematic especially to those living in a ‘very secluded part of the *barangay* (community) where there are no means of transportation’ (Mother of 2, age 23). Most VHCs relayed that they did not have their own vehicle, ‘the only option is to walk’ (Mother of 5, age 31) and it would take them ‘an hour just to reach the health centre’ (Mother of 2, age 22). Respondents also conveyed that rough or elevated roads are still the norm in rural areas and that walking these roads while carrying their children presents a heavy burden.

Respondents also recalled a volcanic eruption in early January 2020 as not only disrupting their children’s vaccination schedule but also affecting their daily routine as local authorities recommended to stay at home or wear masks due to widespread fallout of volcanic ash. Respondents described how families were displaced to an evacuation area (1–2 hours from the health centres), so acquiring successive vaccine doses proved impossible.

#### Fear of COVID-19 and associated community lockdowns

The COVID-19 pandemic emerged as particularly salient as respondents shared that they were obligated to wear face masks and face shields when going outside, especially when going to public places such as health centres, which contributed to vaccination delays and refusals. Some respondents argued that they would just wait for the lockdown policy to be lifted to catch up with their children’s vaccination schedule.

Respondents also shared fears regarding how vaccines, namely polio, were being administered by HCWs amid the pandemic. Respondents explained how the polio vaccine is commonly packaged in small plastic vials from which HCWs squeeze the vaccine directly into the child’s mouth, which is perceived as risky, especially in times when the SARS-CoV-2 virus is widespread. In addition, respondents expressed reservations regarding house-to-house polio and measles vaccination campaigns often viewed as unhygienic, as ‘vaccines should only be given at the health centre and this way of administering the vaccine in the community might lead to contamination’ (Mother of 9, age 43).

## Discussion

This study explored how VHCs and their families in rural and urban communities in the Philippines perceived vaccines in general, as well as their reasons for delaying or refusing childhood vaccinations. Our findings highlight that the caregivers’ childhood vaccine refusal and delays on individual and interpersonal levels are mostly anchored in past experiences and resulting fears (eg, previous experiences of side effects or exposure to Dengvaxia), or that vaccines, similar to other medical technologies, are perceived to contradict cultural beliefs, religious or medical traditions, or the decisions of household heads or other community members. On health system and superstructural levels, poor patient−provider relationships, overwhelmed health facility structures, and barriers associated with COVID-19 or natural catastrophes were described as driving factors for caregivers’ VH.

Our findings mirror evidence from previous studies, including a systematic review and meta-synthesis^13^ surrounding childhood VH from the perspectives of parents, which highlighted that VH is often primarily driven by: (1) risk conceptualisation (ie, toxicity of vaccine ingredients); (2) mistrust (ie, towards both health and pharmaceutical institutions); (3) alternative health beliefs (ie, vaccines viewed as ‘unnatural’); (4) philosophical view (ie, burden of decision making) and (5) lack of information. The finding that delay or refusal of vaccines is not exclusively attributable to firm personal beliefs is mirrored elsewhere.^50^ While previous studies comprehensively laid out determinants associated with VH, these were mostly drawn from HIC pespectives.^10 17 18^ In Australia, for example, Helps *et al*^51^ described that non-vaccinating parents did not have firm intentions to reject vaccines, but were driven by series of events that led them to make a negative decision (eg, HCWs dismissing their concerns about experienced adverse events). In our study, we add to the LMIC perspective by replicating certain findings from HICs, such as the relevance of vaccine safety concerns and mistrust in government vaccine programmes, but also by highlighting factors previously discussed, such as caregivers’ geographical inaccessibility to health facilities and unreliability of vaccine services.^13 52^ Our findings also emphasise conflicting priorities (ie, work, hectic household tasks) that hinder caregivers’ compliance to vaccination schedule, which echoes other findings from LMICs.^53 54^ The relevance of religious and cultural notions is similarly echoed in studies in Indonesia and Malaysia wherein parents expressed a lack of confidence in modern medicine and HCWs, and an understanding that vaccines have impure contents, thus contraindicating religious practices.^55 56^

Previous studies have highlighted how mothers are often responsible for the child’s health, but in many settings can lack the decision-making power to enact what they perceive as being beneficial due to gender norms and power inequities.^57^ Quantitative studies have also argued that religious, cultural, and gender factors are among the three most prominent reasons for individual VH.^58^ Our findings add to this discourse by highlighting how these beliefs are embedded into the broader socio-ecological context, often exacerbated by strong community influences and a lack of health centre accountability. Further analysis of the decision-making processes and how power is exerted by household members and other stakeholders could provide insights that allow for the development of interventions not only targeted at the different decision-makers, but also acknowledging the interplay between the various role-specific socioecological factors.

Our findings also emphasise that caregivers perceive HCWs as dismissive, unavailable or disrespectful in terms of answering questions or concerns about vaccines. This in turn leads caregivers to rely more heavily on other channels for information, such as their own social circle or social and traditional media. However, these channels can function as ‘echo chambers’ (defined ‘as environments in which the opinion, political leaning or belief of users about a topic gets reinforced due to repeated interactions with peers or sources having similar tendencies and attitudes’,^59^ p.1) wherein caregivers are repeatedly confronted with heavily biased information. Social media platforms have proven particularly fertile ground for creating and promoting unverified information that later becomes viewed as fact.^11 12 59 60^ In a large cross-country study, Wilson and Wiysonge^61^ found a significant relationship between social media disinformation campaigns and declining vaccination rates. Recent studies mirror our findings that the magnified misinformation and disinformation received from these social media platforms led to caregivers’ vaccine confusion.^62–64^

The prominent role of fears resulting from the Dengvaxia controversy found in our study highlights a critical need to further explore how vaccine scares can shape narratives about vaccines and health systems. In our case, fears of side effects of ‘new vaccines’ (ie, Dengvaxia) had spilled over to other childhood vaccines, impacting immunisation efforts nationwide. Research among parents of Dengvaxia-vaccinated children has found that parents view the vaccine as causing more harm than good and laying bare the government’s carelessness in implementing vaccination programmes.^31^ Similarly, Migriño *et al*^29^ conducted a survey of two urban communities in the country’s capital, Manila, and found that parental refusal to at least one childhood vaccine was linked to the negative media information and safety concerns related to the dengue vaccine. Our results give voice to the caregivers themselves and highlight how the mental trauma of vaccine scares remains within the family and has profound and persistent effects, which has also been described in the Philippines and elsewhere in relation to fears brought by misinformation to vaccine.^31 65 66^

For the last decade, a considerable amount of literature has been published on how to address VH.^67 68^ Particulary, Dubè *et al*^69^ have argued for the integral need to devise and optimise risk communication strategies to reach and cover the population and capture public interest in simple, empathic and provaccine messaging. This importance of ongoing training for HCWs to be able to communicate and reach people effectively is also resonated by other authors.^70 71^ Risk communication skills that allow for rebuilding trust and transparent and clear health education campaigning aimed at VHCs would be of great importance for reviving vaccine uptake.^72^

This study has limitations. First, VH is a highly politicised topic in the Philippines; social desirability bias therefore may have prevented respondents from disclosing certain reasons for vaccination delay or refusal. Second, due to the COVID-19 pandemic, data collection had to be conducted online. Although tools and procedures were adapted accordingly and efforts were made to minimise selection bias, respondents with weak internet connection or network and those without the necessary devices may not be equally represented. Third, the Philippines is a unique case with regards to VH, both due to the recent Dengvaxia controversy and the particular role of social media in the country,^28 37^ making this setting an enticing case study to understand barriers for vaccination uptake across all socioecological levels, but meriting caution when making broad comparisons to other settings.

## Conclusion

In this article, we give voice to Filipino VHCs who described several overarching concerns regarding childhood vaccines. Applying the SEM framework, our results suggest that, although there are greater structural and contextual forces, individual-level and interpersonal-level concerns are the most salient factors that often inhibit the success of vaccination campaigns. Various strategies and policies to cope with past and current vaccination challenges to bolster vaccine uptake are needed to address individual concerns of VHCs at the centre of any intervention.

## Data Availability

All data relevant to the study are included in the article or uploaded as online supplemental information. The datasets generated in this study are not publicly available due to sensitive and personal nature of the information. Data may be available upon request to authors, with restrictions following ethical approval.
